# Supplementary material for: “My drinking was way worse during the pandemic”: A qualitative analysis of contextual and individual factors impacting alcohol use during the COVID-19 pandemic
Source: PLoS One. 2025 Apr 2;20(4):e0319977. doi: 10.1371/journal.pone.0319977 (PMC11964242; doi:10.1371/journal.pone.0319977)
Supplement: S1 Table — (DOCX) [file pone.0319977.s001.docx]

# Supplementary Table 1 Summary of identified themes and exemplar quotes.

| **Theme** | **Exemplar Quotes** | |
| --- | --- | --- |
| **During the COVID-19 pandemic, participants encountered fewer barriers to alcohol use resulting in changes to their drinking patterns** | | And it wasn't just, you know, like us shutting down. You know, I work kind of in sales so all my customers kind of stopped working too. So it was like, oh I have a lot of freedom because I don't even have anyone calling me for work <laughs> at all because they're not working either. (PM_06, 36-year-old Hispanic White female with severe AUD symptomology)    Yeah, you know what, that's good. All that, and between the ankle, celebrating, not having the meetings that I had been going to because of the pandemic-- I'm sure all that kind of contributed to me just taking off. (PM_10, 63-year-old non-Hispanic White female with severe AUD symptomology)  I mean, I don't drink hard liquor. But I do like my beer. And initially, I found it a lot easier probably to have more than I normally had, especially in the Year 2020. And I don't-- I mean, I don't get to the point where I fall over drunk or anything, but I do have a few. And it's just kind of relaxing thing, and I do work out a lot and it's kind of one of my rewards that I guess give to myself. (CM_06, 54-year-old non-Hispanic White male with no AUD symptomology)  I think people just wanted to drink to maintain kind of like a sense of normalcy in a way. But because it was like less limited by other factors I think we were just drinking more than usual. (PM_15, 24-year-old non-Hispanic White female with moderate AUD symptomology)  I think just like the accessibility of it, knowing like “Oh, let me just walk two feet, pour myself my own drink. There’s no bar tab that I can see,” and not having to like get up in the morning, get the kids to school or when they were in school, take them to school and then I could just go back home. I didn’t have to go into the office. There wasn’t as much stuff going on, especially when they didn’t have like sports or anything. My kids have like three activities a day. So, to go from like 100% to like nothing, I was like “Wow, nothing else to do,” and like everyone else was bored. So, they’d be like “Oh, I’m going to come over and sit on the porch and drink with you,” and I’m like “Great. That’s what we’re going to do. Nothing else to do.” (PM_11, 37-year-old non-Hispanic White female with severe AUD symptomology) |
| **During the COVID-19 pandemic, the availability of alcohol facilitated alcohol use.** | | Interviewer: Did you have alcohol delivered during the pandemic?  Participant: Oh, I didn't have it delivered, but I could order it online and go pick it up.  Interviewer: Okay. And I'm guessing-- was that something new? Had you done that before the pandemic?  Participant: Yeah, that was new. That's like, oh, I can do a whole shopping cart? You know, like even I can browse the thing. It's so convenient, and like membership discounts, you get this. But then also planning to go pick it up. Yeah. (PM_12, 47-year-old non-Hispanic White male with mild AUD symptomology)  Participant: Oh, yeah. My drinking was way worse during the pandemic than it is now.  Interviewer: Yeah. How do you understand that? What’s contributed...  Participant: Well, it was more available because I know here in Texas, the Governor had approved for alcohol to be delivered to the house. Like, if you would order it over the phone, it could be delivered during COVID. So, I would utilize that sometimes. (CM_11, 33-year-old Hispanic White female with severe AUD symptomology)  Interviewer: Was it easy or hard to get alcohol during the pandemic for you? Or was it about the same? Participant: It was easier. I mean, you have so many places, like, selling to go slushies in a bag from a bar. <laughs> Like, I mean, come on, now. (CM_07, 38-year-old non-Hispanic White female with moderate AUD symptomology)  Interviewer: Did it become easier or harder to get alcohol during the pandemic or no?  Participant: I feel like it was easier because they had delivery service… (CM_02, 31-year-old non-Hispanic Black female with severe AUD symptomology)  …*There’s a liquor store almost everywhere.* *There’s a beer store almost everywhere.* It’s easy to get and you can even order it for delivery as well. So, I think that’s the reason why it became alcohol and nothing else and because it’s legal too. So, I can’t smoke weed because of all this other stuff, but I know a lot of folks who use substances like that, but *alcohol just became something that was available and easy to get.* (PM_02, 36-year-old non-Hispanic Black male with no AUD symptomology) |
| **During the COVID-19 pandemic, alcohol became more easily accessible which fostered changes in alcohol use patterns among participants.** | | Yeah, no it changed like the convenience and right there. And it’s sort of-- I was looking for an outlet, and that was the easy hook for me. Okay, go to the fridge, get it. And I know over time if I'm looking at this, like I would start craving when is this thing over so I can go get this thing. (PM 02, 36-year-old non-Hispanic Black male with no AUD symptomology)  I think just like the accessibility of (alcohol), knowing like “Oh, let me just walk two feet, pour myself my own drink. There’s no bar tab that I can see,” and not having to like get up in the morning, get the kids to school or when they were in school, take them to school and then I could just go back home. I didn’t have to go into the office. There wasn’t as much stuff going on (PM 11, 37-year-old non-Hispanic White female with severe AUD symptomology)  We can drink as much as we want. And I know the wine rack was always full during those early parts of the pandemic, which was great, but also, I definitely was drinking more. (PM_15, 24-year-old non-Hispanic White female with moderate AUD symptomology)  …you know, before when there was no to go slushy, like, I wouldn't have even thought of one. But then they're, like, sitting there on a table, if I want one, I'm going to get one, you know? Just having those types of things there I'm more likely to buy them or get them or do that. (CM_07, 38-year-old non-Hispanic White female with moderate AUD symptomology)  …because both of our parents are or were recovering alcoholics we typically don't keep-- we just don't keep alcohol in the house, whereas during this time, 2020, '21, we did keep beer and wine and some liquor in the house. (PM_05, 40-year-old non-Hispanic Black female with mild AUD symptomology) |
| **Participants described drinking as a way to relax, unwind, and escape the chronic stress, anxiety, boredom, isolation, and negative emotions during the COVID-19 pandemic** | | I don't know, I feel like a lot of drinking, especially during COVID, was just out of boredom, like being home and being bored. (PM 11, 37-year-old non-Hispanic White female with severe AUD symptomology)  I felt like I was on vacation, but it wasn't a happy vacation…when I started drinking my heaviest I was just kind of like, well, I don't have to go anywhere. Well, I don't care. Well, I had a rough day. I'm going to do it. And so it was kind of like this like I'm in a really bad place….I wasn't like drinking a bottle of wine a day type of a situation, but I would have a beer while I was like unwinding watching a show at the end of the day. And I didn't think about it at all. I just did it during that time. (PM 08, 31-year-old non-Hispanic White female with no AUD symptomology)  Well, it definitely helped me to relax. I think on this weird level there was kind of like this feeling that I didn't have control over anything, anything…. I can go to the liquor store, and I can pick out my own beer. And, you know, I got everything else taken out from under me, but I can do that. And then I can have a drink on a Monday night and nobody's going to tell me I can't. So it was bad, I think. (PM 08, 31-year-old non-Hispanic White female with no AUD symptomology)  I was noticeably drinking more and especially because things were stressful working in healthcare during those times. Our staffing was crummy…after a hard day you want a glass of wine with dinner to wind down and it wasn't usually more than a glass or two, but it was like almost at--it was like five or six days a week. So if I opened a bottle last night and had two glasses, then the next day, I had to finish the bottle with another two glasses. And then it was kind of--it wasn't like that every single week, but it was definitely more frequent and noticeable. (PM 15, 24-year-old non-Hispanic White female with moderate AUD symptomology)  Interviewer: What did you do for Christmas?  Participant: I was sitting in my condo just getting drunk.  Interviewer: For Christmas?  Participant: Christmas at home drinking.  Interviewer: Christmas at home drinking.  Participant: Yep, absolutely alone. (PM 10, 63-year-old non-Hispanic male with severe AUD symptomology) |
| **Participants described drinking as a way to reward themselves and to experience positive emotion and pleasure in a context with reduced opportunities for rewarding activities** | | I think in a positive light, it’s been a way to feel like I can still have a treat…I can’t go out to restaurants, I can’t see friends, but it’s like you know what, I can have a glass of wine and I can watch a movie, and that is a nice way to feel like I'm taking care of myself…It’s just like it’s something nice I feel like I can do for myself that’s relatively low impact. (PM 13, 25-year-old non-Hispanic White female with no AUD symptomology)  During COVID, the beer became a primary focus. I sat on my couch and really focused on every sip because it was a sensual experience that I wasn’t getting anywhere else. (CM 06, 54-year-old non-Hispanic White male with no AUD symptomology) |
| **Participants described drinking more heavily during social events, and drinking alcohol as a way to celebrate (renewed) ability to spend time with friends and family** | | I think we celebrated bigger, like all in celebrate. So, yeah, I would say that happened, like, because now there's always a reason to celebrate, which is cool, and we always are good-- we love a good celebration. But it felt like, ooh, we can really celebrate this time. We can be somewhere. We can do something about how we feel about this thing and so, yeah, we went in hard for the celebrations that were-- that we could do. So it was cool. It was cool to me because it's like, "Yeah, I get to celebrate and everybody is celebrating and we're all enjoying it." And I think it just kind of threw more into it because we couldn't celebrate for so long. And then now that I can celebrate, like, yeah, let's really get it in. (CM 01, 44-year-old Hispanic Black female with no AUD symptomology)  So, here again, it's always about having a good time, the celebrating of something. You make up “Okay, we’re going to celebrate the fact that I haven’t seen you since last year,” just the celebrating of the whole deal when you got together. (CM 03, 66-year-old non-Hispanic Black female with severe AUD symptomology)  "We also went for a week on Martha’s Vineyard and we had friends that rotated in and out of the house that we rented and like drank more then. So, it was like COVID-related in the sense that we were back being able to do these things, but like it wasn’t drinking out of like boredom or sadness. It was drinking out of like “Woohoo, we’re back. We’re celebrating,” and then it’s like “Oh, my god, wait, why did I drink so much?" (PM 11, 37-year-old non-Hispanic White female with severe AUD symptomology) |
