# Supplementary material for: “My drinking was way worse during the pandemic”: A qualitative analysis of contextual and individual factors impacting alcohol use during the COVID-19 pandemic
Source: PLoS One. 2025 Apr 2;20(4):e0319977. doi: 10.1371/journal.pone.0319977 (PMC11964242; doi:10.1371/journal.pone.0319977)
Supplement: Attachment — Submitted filename: Response to Reviewers.pdf [file pone.0319977.s003.pdf]

ARG
ALCOHOL RESEARCH GROUP

December 04, 2024

Dear Dr. Jerolmack,

My co-authors and I appreciate your consideration of our manuscript. After carefully considering the reviewers' feedback we have made the requested changes to the manuscript entitled *"My drinking was way worse during the pandemic": A Qualitative Analysis of Contextual and Individual Factors Impacting Alcohol Use During the COVID-19 Pandemic*. We feel the paper is stronger after incorporating the reviewers' suggested edits.

We appreciate the opportunity to revise this manuscript and hope that it will be considered for publication in PLOSE ONE. We are happy to nominate additional reviewers if required.

Thank you in advance for your consideration.

Sincerely,

Anthony Surace

Anthony Surace, PhD
Associate Scientist

Reviewer #1: Thank you for sharing this manuscript. The main claims of the paper are that contextual and individual level factors such as reductions in barriers to alcohol use, increases in alcohol availability, and increases in alcohol accessibility contributed to increased alcohol use during the COVID-19 pandemic. These environmental factors interacted with personal emotions to increase consumption. These claims are significant given the limitations of quantitative investigations of increased alcohol use, which have not addressed motives and rationales.

The main strengths of the paper are its methodological rigor, contextual focus, and rich data. The qualitative approach is appropriate for exploring in-depth rationales, motivations, practices, and beliefs that led to increased alcohol use. The analysis effectively captured contextual factors unique to the pandemic which provides insights into the complex interplay between environmental conditions and individual behavior. The detailed quotes from participants offer vivid illustrations of their experiences.

The main limitations of the paper are incomplete analyses of the data, lack of theoretical distinction between the environmental factors, and an insufficient literature review. See below for details on specific areas for improvement:

1. The literature review could be developed, especially as it pertains to overall public health or social science literature published during the pandemic. It could start broad and then delve into the particular issue of alcohol use. The literature review is also lacking in historical context. Comparisons could be made to previous crises or emergencies, which would provide a broader perspective on how the pandemic's impact on alcohol use compares to other major events. Additionally, the literature review could address social isolation and social reinforcement which seem to be key to the analysis.

> Response: We thank the reviewer for this comment. We have added text reviewing the literature on changes in alcohol use post-disasters to our introduction as well as social isolation in the context of COVID-19. This includes a comparison of how COVID-19 may have impacted alcohol use in different ways due to its unique nature (see lines 68-84).

2. Lines 90-91 mention that the limits of other qualitative research is that it is restricted to specific populations, but your population is restricted as well (it is not representative). I think this should be stated more explicitly.

> Response: We agree with the reviewer- our sample was quite specific. We have edited the manuscript to note this and have removed this limitation from our description of past literature (see lines 105-122).

3. Lines 110-119 mention surveys conducted in the past. This manuscript could draw out the differences between the surveys done and your interviews a bit more.

> Response: We have added a description outlining the differences of the previously cited survey studies to the Introduction (see lines 123-136).

6001 Shellmound St., Suite 450
Emeryville, CA 94608-1010
T: (510) 898-5800 | **F:** (510) 985-6459
www.arg.org | info@arg.org

4. The methods section does not state a response rate.

> Response: We have included the response rate for the qualitative interviews in the Methods section (see line 162).

5. Lines 190-192 restate the three environmental factors which contributed to increased alcohol use (reduction in barriers, increased availability, and increased accessibility). It is not immediately clear to me how different these factors are. Accessibility and availability of alcohol increase seem particularly similar to me. Can you draw out the differences?

> Response: We agree that the difference between availability and accessibility can be difficult to distinguish and the importance of having these clearly defined and differentiated. We have more clearly defined these themes on lines 216-222.

6. One of your arguments is that participants were more likely to drink a lot at home versus at bars. Participants likely drank at home before the pandemic as well, was it always more at home? Generally, I am trying to understand what is different about being at home during a pandemic versus these participants being at home for other reasons. When they had long weekends or breaks from work did the same patterns in alcohol use arise? I'm not sure how "always having beer in the house" or "having alcohol in their homes" during the pandemic was different than the alcohol in the house beforehand? What's special about the pandemic here?

> Response: We agree with the reviewer that more information is needed to distinguish between participants' drinking behavior pre- and during the pandemic. We have created an additional table ("Table 2 Participant Alcohol Use") which highlights how participants' drinking changed, including the frequency of self-reported alcohol use while at home (which increased during the pandemic). However, it is beyond the scope of our data to determine how extended periods at home (e.g., "long weekends") pre-pandemic may have influenced alcohol use.

7. Lines 253-256 mentions "socially reinforcing their shared change in alcohol use." Why might this have happened? Did others experience a similar reinforcement? Why were there a lack of work obligations for people who didn't lose their job? Why were there greater workloads on-site, if that was the case?

> Response: We thank the reviewer for these questions- these are important things to consider when analyzing the data. We expanded the Discussion section to include a brief discussion of potential reasons for this social reinforcement (see lines 618-632). Unfortunately, we did not ask participants about the specifics of their work obligations, so it is beyond the scope of these data to address potential differences in employment responsibilities and alcohol use.

8. Besides delivery services and to-go drinks, were there other things that changed availability? You also mention this availability lowering social constraints/making it more socially acceptable but don't flesh out an argument for this. There is a lot more that could be said about social pressures and shifting norms, and the mechanisms by which it became more socially acceptable.

Response: Reviewer 1 brings up some interesting points with this comment. We agree that there were likely other forces driving changes in alcohol availability during the pandemic, However, we are limited by our data- alcohol delivery and to go drinks were the examples of increased alcohol availability shared by participants during our interviews. They did not share other ways that availability may have been increased. Similarly, only one participant implied that the increased availability of alcohol may have impacted social acceptability of alcohol use. Given this theme did not reach saturation, we feel it would be inappropriate to speculate on the potential impact of social pressures/norms of alcohol use in this manuscript. We acknowledge these limitations and advocate for further work to be done in these topic areas (see lines 726-745).

9. The point the interviewee makes about relaxation in lines 370-377 could be fleshed out more. Seems an important argument about the stress of the pandemic creating a desire for relaxation mechanisms. So it might not just be accessibility of alcohol making it an “attractive option for stress relief,” but the pandemic itself (I know you go more in depth on this in theme 4, so this quote could be moved to that section)

Response: We agree with the reviewer and have moved this quote and discussion to the theme four section (see lines 437-454).

10. For theme 4, can you spell out the negative emotions? You jump into stress and boredom right away, but it is unclear what the range of emotions described by participants are.

Response: We have spelled out the negative emotions expressed by participants on lines 432-434.

11. The ideas about control are really interesting. The manuscript could emphasize this and analyze the quotes further. Control seems qualitatively different than boredom, for example, and could use its own analysis.

Response: We agree that this is a fascinating topic. We reviewed the interview transcripts and unfortunately this was the only instance where this topic emerged. In hindsight we should have asked participants about this, but it was not something we had considered when we were conducting these interviews. We acknowledge this limitation in the manuscript see lines 727-730.

12. Theme 5 is too short relative to the other sections.

Response: We have expanded the section on theme 5 including a more detailed description of participants’ use of alcohol (see lines 530-566).

13. Social isolation comes up in the discussion but not as much in the results. Any more quotes or narratives that highlight this social isolation? Also, there is a lot of literature on this that could be included in the literature review.

> Response: We have expanded the theme 4 results to include quotes relating to social isolation among participants and its impact on their alcohol use (see lines 499-529)

14. In the limitations section, it would be helpful to mention what kind of workers you interviewed. It seems work arrangements are an important aspect to bring up as part of the analysis. How did their work situations uniquely contribute to your findings? The demographics table could include work arrangements as well.

> Response: We agree with the reviewer that participants' employment likely impacted their behaviors during the COVID-19 pandemic. Unfortunately, we have limited data regarding their specific jobs and work arrangements- although some participants mentioned their occupation it was not a standard survey/interview question. We are therefore limited in our ability to offer detailed descriptions of participants' jobs/work arrangements. We have expanded the demographics included in table 1 to include more detailed employment information (e.g., the number of participants who self-identified as essential worders during COVID). We have also expanded the limitations section to reflect this (see lines 726-745)

I enjoyed reading this paper and I think it makes important contributions to our understandings of alcohol use.

> Response: We thank the reviewer for this positive comment and for the constructive critiques that have helped to improve the quality of the manuscript.

Reviewer #2: This article analyzes the contextual and individual-level forces that motivated increased alcohol use during the first year of the COVID-19 pandemic. Using qualitative interviews with 26 participants, it describes how changes in the availability and accessibility of alcohol interacted with social isolation, negative emotions, and lack of routines to make alcohol a more recurrent and acceptable coping mechanism.

The paper is clear, well written, and adequate in its use of evidence to support its claims. My comments for revision are related to (1) strengthening the statement of motivation for the study; (2) providing more details on the sample and how they affect the paper's results, (3) clarifying the contributions.

1. The article currently justifies its methods and approach by stating that there is little qualitative research on alcohol consumption during the COVID pandemic and that the existing qualitative research is limited due to its "focus on specific populations." Yet this article hardly remedies that, since its sample is small (n=26) and comprises a very specific population as it's derived from the NAS-C19 survey, thus limited to people who have reported drinking above NIAA guidelines. Plus, the sample is heavily made of White, college-educated participants. I am not suggesting in any way that this compromises the article's results, but to motivate the paper based on the

6001 Shellmound St., Suite 450
Emeryville, CA 94608-1010
T: (510) 898-5800 | **F:** (510) 985-6459
www.arg.org | info@arg.org

sampling limitations of previous qualitative research while exhibiting these same kinds of limitations is not the best approach. Likewise, to say that “Another limitation of qualitative work to date has been its use of open-ended survey responses to assess motives for drinking which severely limits the amount and depth of qualitative data” (lines 97-98) strikes me as odd, since survey studies are not qualitative.

Response: We agree with the reviewer. In hindsight, our sample was quite specific. We have edited the manuscript and have removed this limitation from our description of past literature. We also agree that survey studies are not qualitative. The cited studies treated responses to open-ended survey questions as qualitative data. We added further details about why this is a limitation of the literature (see lines 123-136).

There are several demographic characteristics in the sample that either need to be expanded on or added to the table. Among the ones I suggest expanding on are:

a. Employment status: at several points in the article we read about work-related obligations or lack thereof, or unemployment, impacting alcohol consumption (i.e. line 238). How exactly does this variable mediate the patterns the authors find? Did any of these people lose their jobs during the pandemic?

Response: We agree with reviewer 2 that employment status is an important factor in these analyses. We have expanded table 1 to include more details about participants’ employment (e.g., if they became unemployed during the pandemic. We note, however, that it is beyond the scope of these qualitative data to conduct mediation analysis.

b. Income: from Table 1 we learn that about half of the sample earns: $70,000 or more. Is this individual or household income? What about the rest?

Response: We have clarified in table 1 that this is referring to household income. $70,000 was the median household income for the sample.

c. Did people with Alcohol Use Disorder Symptoms (57.7% of the sample) differ from the rest in the patterns identified?

Response: We did not observe notable differences in our qualitative analyses (i.e., theme frequency) based on participants’ AUD symptomology scores. We have now included categorical AUD severity scores to participant information post quotes.

Two characteristics that are not included in Table 1 and I suggest including:

a. Children. Several times we read about children in participants’ quotes (line 207; 246-247; 410) and it seems like both presence and absence of children act as motivations for drinking. I suggest working this into the analysis.

Response: We agree with reviewer 2 that given childcare came up during the interviews that that the number if minors living in participants' households is important information to include. We have therefore added it to table 1.

b. Geographic location. We know nothing about where participants live, which seems important because some contextual factors could be shaped by different local policies (i.e., stay-at-home orders; allowing bars to remain open; alcohol delivery).

Response: We agree with reviewer 2 that contextual factors could shape alcohol policies. We have therefore added geographical location to table 1.

3. Perhaps related to my first point about the motivation for this article, I'd suggest highlighting its contributions a bit more. For instance, you could comment on whether previous studies differentiate between contextual and individual level factors in participants' alcohol use. Or, and this might be beyond the scope of this analysis, you could do more with a theme I find really interesting which is how the interaction of contextual and individual factors lead to a change in perceptions of social acceptability in alcohol use. In the discussion (lines 520-523) it "unclear when alcohol could or should be used." It seems like this is a novel contribution of this study that could be highlighted.

Response: We have expanded our Discussion section to highlight our contributions to the field more see lines 770-772. We agree with reviewer 2 that the interaction of contextual (e.g., alcohol availability) and individual factors (e.g., perceived social norms) may have impacted alcohol use. However, only one participant described this phenomenon. Given this lack of saturation of the theme, it is beyond the scope of the data to speculate on the potential impact of social pressures/norms of alcohol use in this manuscript. We acknowledge these limitations and advocate for further work to be done in these topic areas (see lines 726-745).

6001 Shellmound St., Suite 450
Emeryville, CA 94608-1010
T: (510) 898-5800 | **F:** (510) 985-6459
www.arg.org | info@arg.org
