# Supplementary material for: “My drinking was way worse during the pandemic”: A qualitative analysis of contextual and individual factors impacting alcohol use during the COVID-19 pandemic
Source: PLoS One. 2025 Apr 2;20(4):e0319977. doi: 10.1371/journal.pone.0319977 (PMC11964242; doi:10.1371/journal.pone.0319977)
Supplement: Attachment — Submitted filename: Response to Reviewers_2.0.pdf [file pone.0319977.s004.pdf]

January 27, 2024

Dear Dr. Jerolmack,

My co-authors and I appreciate your consideration of our manuscript. After carefully considering the feedback we have made the requested changes to the manuscript entitled *“My drinking was way worse during the pandemic”: A Qualitative Analysis of Contextual and Individual Factors Impacting Alcohol Use During the COVID-19 Pandemic*.

We appreciate the opportunity to revise this manuscript and are grateful for your willingness to accept the paper after the noted revisions in PLOSE ONE.

Thank you in advance for your consideration.

Sincerely,

Anthony Surace

Anthony Surace, PhD
Associate Scientist

1. The reviewer points out a small discrepancy between reporting and sample; please reconcile

> Response: We thank the reviewer for pointing this out. The number we had previously added was the average number of children in the household (not the frequency of participants with children in their household). We have edited table 1 to make this more explicit.

2. I think you need to say a bit more about the potential problems of self-reporting alcohol use. I appreciate that you get at some of the issues in the "limitations" portion of the paper, but the influence of affective states on reporting of alcohol isn't the only potential issue. We can imagine how selective or inaccurate recall may play a role, or social desirability bias, etc. And the issue of recall presumably grows the longer in time you ask people to look back upon--e.g., a survey asking how many drinks you had YESTERDAY is likely more accurate than one asking in the past WEEK, which in turn is better than last MONTH, and so on. I understand you are more interested in patterns [drinking "more"] than specific numbers of drinks, but the same kind of issues may apply. There must be some studies that try to gauge the accuracy of self-reporting on alcohol consumption that can help us understand how concerned we should be, no?

> Response: We thank the editor for their feedback. We agree that retrospective self-report methodologies have inherent limitations including recall biases which can impact accuracy of data collected. We have expanded the limitations section to acknowledge this. See lines 747-775.

3. Relatedly, and given that these are accounts, you need to go through the entire ms. and add qualifiers such as "reported" or "said" in front of descriptions of actions if they are based on self reports. For instance, "evidences shows that people who SAID THEY used more alcohol."

> Response: We thank the editor for pointing this out. We have added qualifiers to description of alcohol use in the manuscript specifying that these reflect self-reported behaviors. For example, see lines 294, 401, and 601.

4. Also related to the issue of accounts, I would like you to be more careful about talking about MOTIVATION. I understand that this is the way people talk in this field, but it's very hard to ever get at motivation and is made all the harder when one is using one-off interviews in which people are asked to look back. Can we really say that we have captured motivation when we have no T1 to compare to T2? What we actually have are cross-sectional data [the one shot interview] in which you are relying on accounts to construct a narrative in which you can say that event or mood at T1 caused behavior at T2. I leave it up to you how to deal with it, but I advise being more careful about confidently stating claims about motivation.

> Response: We thank the editor for their feedback. We have expanded the limitations section of the manuscript to reflect this feedback (see lines 773-78). We have also hedged some of our previous statements regarding alcohol use motivations (e.g., lines 30, 601 and 780).

ARG ALCOHOL RESEARCH GROUP
6001 Shellmound St., Suite 450
Emeryville, CA 94608-1010
T: (510) 898-5800 | **F:** (510) 985-6459
www.arg.org | info@arg.org
